# Rotenone and Paraquat Linked to Parkinson’s Disease: Human Exposure Study Supports Years of Animal Studies

**DOI:** 10.1289/ehp.119-a259a

**Published:** 2011-06

**Authors:** Angela Spivey

**Affiliations:** **Angela Spivey** writes from North Carolina about science, medicine, and higher education. She has written for *EHP* since 2001 and is a member of the National Association of Science Writers

A growing body of evidence suggests pesticides may play a role in Parkinson’s disease (PD) in humans. Self-reported PD has been associated with lifetime use of pesticides, and animal studies have suggested that the pesticides paraquat and rotenone can cause oxidative stress and mitochondrial dysfunction, respectively—posited mechanisms of action in PD—as well as symptoms in rodents similar to human PD. Now, researchers have linked human exposure to paraquat and rotenone with PD **[*EHP*** **119(6):866–872; Tanner et al.]**. Their study is the first analysis of pesticides classified by presumed mechanism of action rather than by intended use or chemical class.

The researchers assessed lifetime use of pesticides as reported by participants in the Agricultural Health Study, a prospective study of private pesticide applicators (mostly farmers) and their spouses in Iowa and North Carolina. In the nested case–control study, neurologists specializing in movement disorders identified 110 people with PD and 358 controls without who were frequency matched by age, sex, and state.

The researchers collected detailed information about use of 31 different pesticides as well as covariate information including lifelong smoking and family history of PD. Each pesticide was classified according to its mechanism of action as either an oxidative stressor or a mitochondrial complex I inhibitor. For participants with PD, the researchers considered pesticide use that occurred before their PD diagnosis. For controls, they considered pesticide use that occurred before the median age of PD diagnosis for cases in the corresponding age-, sex-, and state-specific groups.

Among the mitochondrial complex I inhibitors studied, the researchers found the strongest association between PD and use of rotenone. Among oxidative stressors, they found the strongest association between PD and use of paraquat. Participants with PD were 2.5 times more likely than controls to have reported use of rotenone or paraquat.

The results may have far-reaching implications, considering the widespread use of these pesticides. Paraquat remains one of the most widely used herbicides worldwide, and rotenone was used ubiquitously before most uses were voluntarily stopped in the United States in 2007. Other agents associated with mitochondrial complex I inhibition, such as permethrin, remain in common use.

The authors point out several limitations of the study, including the fact that participants reported exposure to many pesticides, and effects of other agents can’t be excluded. However, the associations between PD and paraquat and rotenone remained after adjustment for overall pesticide use. It also was impossible to measure pesticide exposure, only estimate it retrospectively. Future investigations of combinations of pesticides and of other mechanistic groups will be important, as will mechanistic studies that model exposure conditions similar to those occurring in humans.

